# Bone metastasis risk factors in breast cancer

**DOI:** 10.3332/ecancer.2017.715

**Published:** 2017-01-24

**Authors:** Catarina Pulido, Inês Vendrell, Arlindo R Ferreira, Sandra Casimiro, André Mansinho, Irina Alho, Luís Costa

**Affiliations:** 1 Serviço de Oncologia Médica, Hospital de Santa Maria, Centro Hospitalar Lisboa Norte, Avenida Professor Egas Moniz, 1649–035 Lisboa, Portugal; 2 Luis Costa Lab, Instituto de Medicina Molecular, Faculdade de Medicina da Universidade de Lisboa, Avenida Professor Egas Moniz, 1649–028 Lisboa, Portugal; * These authors contributed equally to this work

**Keywords:** bone metastases, breast cancer, risk factor

## Abstract

Bone is the single most frequent site for bone metastasis in breast cancer patients. Patients with bone-only metastasis have a fairly good prognosis when compared with patients with visceral disease. Nevertheless, cancer-induced bone disease carries an important risk of developing skeletal related events that impact quality of life (QoL). It is therefore particularly important to stratify patients according to their risk of developing bone metastasis. In this context, several risk factors have been studied, including demographic, clinicopathological, genetic, and metabolic factors. Most of them show conflicting or non-definitive associations and are not validated for clinical use. Nonetheless, tumour intrinsic subtype is widely accepted as a major risk factor for bone metastasis development and luminal breast cancer carries an increased risk for bone disease. Other factors such as gene signatures, expression of specific cytokines (such as bone sialoprotein and bone morphogenetic protein 7) or components of the extracellular matrix (like bone crosslinked C-telopeptide) might also influence the development of bone metastasis. Knowledge of risk factors related with bone disease is of paramount importance as it might be a prediction tool for triggering the use of targeted agents and allow for better patient selection for future clinical trials.

## Introduction

Breast cancer (BC) is mainly diagnosed in early stages (90–95%), however 20–30% of these patients become metastatic [1] and to this day incurable. Bone is the single most frequent site for metastases [1] and is involved in about 70% of all metastatic patients [2, 3]. In fact, up to 13.6% of BC patients diagnosed in stage I-III will develop bone metastasis (BM) at 15 years of follow-up [4]. Although not curable, patients with bone as a single metastatic site have a better prognosis than those with visceral or both bone and visceral disease. In fact, bone-only metastatic BC has a median overall survival (OS) which ranges from 40–65 months [5, 6].

Several factors seem to affect the metastatic pattern, including demographic, clinical, pathological, and genetic factors. In BC, BM present a radiographic pattern that can be lytic, blastic, or mixed. Knowing which factors are associated with the development of BM is of great importance, since it might help to predict which patients have an increased risk of a bone relapse and desirably lead to a tailored therapy. Moreover, this knowledge might enable better selection of patients for future clinical trials. Given the lack of information in this field, we performed a literature review on risk factors for BM in BC patients.

## Pathophysiology of breast cancer metastasis to the bone

BC spread is a complex multistep process. It begins with the epithelial-to-mesenchymal transition (EMT) of locally invasive carcinoma cells which then enter the lumina of blood vessels, a process called intravasation [7–10]. Once in the systemic circulation, these circulating tumour cells (CTCs) must survive a variety of stress factors in order to reach the bone marrow, namely the stress imposed by matrix detachment [11, 12], the shear forces, and the predation by cells of the innate immune system [13]. To evade these threats, CTCs form relatively large emboli via interactions with blood platelets [14–16]. However, the majority become trapped in capillary beds during its first passage through the circulation [17–19]. Nevertheless, some CTCs may avoid this rapid trapping because of their plasticity or chance passage through arteriovenous shunts [20]. Eventually, some CTCs become lodged in the microvasculature of distant organs and initiate intraluminal growth, rupturing the walls of surrounding vessels, and placing cells in direct contact with the parenchyma of a specific organ [21, 22]. Also, CTCs may be able to extravasate from the vessels lumina into the stromal microenvironment by penetrating the endothelial cell and pericyte layers [23].

Yet, as early as 1889, a British pathologist named Stephen Paget noted that the patterns of metastases formation could not be explained either by random scattering throughout the body or by the patterns of dispersal from the breast through the general circulation [24]. He proposed the 'seed and soil' hypothesis and intuited the notion of metastatic tropism. Available evidence suggests that CTCs dispersion is affected by the layout of the vasculature and once they have arrived in these various sites, cancer cells survive and colonise only in those tissues that provide them with specific chemokines, trophic factors, and mitogens [25]. Nevertheless much is still unknown regarding BM formation. Research has focused on identifying determinants of organ-specific metastasis, including intrinsic cancer cell properties, such as genes and pathways regulating colonisation [25–28]. More recently, Hoshino *et al*, demonstrated that tumour exosome integrins prepare a favourable microenvironment at future metastatic sites and mediate non-random patterns of metastasis [29]. Thereby BC metastasis organotropism, tumour-microenvironment interactions, and strategies to address them for prevention and treatment of BM are areas of intense investigation [30, 31]

BC CTCs reach the bone through the vessels feeding the marrow. There, CTCs adhere to specialised stromal cells coating the bone facing the marrow, undergo mesenchymal-to-epithelial transition (MET), and start releasing parathyroid hormone-related peptide (PTHrP) [32]. PTHrP causes nearby osteoblasts to increase receptor activator of NF-?B ligand (RANKL) and decrease osteoprotegerin (OPG) expression [33]. As a result, osteoclast precursors mature in functional osteoclasts that undertake osteolysis causing bone demineralisation and exposing the extracellular matrix within the bone. In this process, transforming growth factor ? (TGF-?), calcium, bone morphogenetic proteins (BMPs), fibroblast growth factors (FGF), and insulin-like growth-factor-1 (IGF-1) are released, enabling cancer cell proliferation and survival [34]. TGF-? fuels further growth of BC cells, inducing them to produce more PTHrP, resulting in a self-sustaining positive-feedback loop called the vicious-cycle of bone metastasis [34].

Even if BC cells preferentially activate osteoclasts, resulting in osteolytic metastasis, osteoblastic areas are in general also present [35]. Yi *et al* followed the course of metastases development from human BC xenografts in nude mice and found that overexpression of tumour-derived platelet-derived growth factor-BB (PDGF-BB) in MDA-MB-231 human BC cells induced partial osteosclerosis in the BM which are usually pure osteolytic [36]. BC cells also produce endothelin-1 (ET-1) that activates osteoblasts, resulting in the accumulation of immature mineralised bone (sometimes termed osteoid), and suppresses osteoclast activity [37]. The totality of the molecular mechanisms underlying this switch from osteolytic to osteoblastic activity is not understood. Nevertheless, it seems clear that the processes of bone resorption and formation are almost always linked (although this coupling may be distorted in cancer), and that osteolytic and osteoblastic metastases belong to the same spectrum of disease. Moreover, it is hypothesised that activated osteoblasts secrete large amounts of growth factors during the construction of mineralised bone and that some of these factors are diverted by cancer cells thereby further increasing their proliferation [38].

## Risk factors for bone metastases

### Demographic and clinicopathological factors

Several studies have been performed in order to determine demographic, clinical, and pathological factors that could be associated with higher risk of BM. Some of these factors are summarised in Table 1.

#### Age

There are conflicting results when analysing the effect of age in the development of BM. Some studies have shown that BC patients who develop bone-only metastasis tend to be older than those who relapse with both visceral and bone disease [3]. This conflict can be, in part, a result of different studies using different definitions of age: age at time of cancer diagnosis, or age at time of metastatic disease diagnosis.

In fact, in a retrospective German multicentre study (n = 226), BC patients older than 65 at diagnosis had a 1.5-fold increased risk of developing bone-only disease when compared to younger women [39]. In this study, when included in a multivariate model (including subtypes of BC, age, histological subtypes, tumour size, number of affected lymph nodes, grade and nuclear proliferation index), age at diagnosis was the second most important factor for the development of BM (χ^2^ = 17) after BC subtype (χ^2^ = 28) [39].

Nevertheless, other studies seem to report a surprising inverse relation between age at diagnosis and the risk of developing distant metastasis, regardless of whether it is bone and/or visceral disease [40]. Purushotham *et al* followed 3552 BC patients over a median 6.32 years and reported that patients above 40 years old at diagnosis had a significant decrease in the risk of developing distant metastasis with increasing age. Specifically, there was a decrease in BM with increasing age (adjusted hazard ratio (HR) of 0.50, 95% confidence interval (CI) 0.31–0.81 for women with 40–49 years old and adjusted HR of 0.37; 95% CI 0.19–0.69 for women above 70 years old) [40]. Worth noticing that in this study, only 24 patients with BM were older than 70 years. Another study, by Liede *et al* with 2097 Canadian patients, also showed that age can be a protective factor for BM as patients younger than 40 had a 2.01 higher risk of developing BM than older women (HR = 2.01; 95% CI 1.40–2.89; p = 0.0002) [4]. While Purushotham *et al* only included patients with BM [40] Liede *et al* included patients with both bone and visceral disease [4]. Therefore the inclusion criteria do not seem to be the reason behind this unexpected inverse relation.

On the other hand, other studies actually failed to identify age as an independent risk factor for BM in BC patients [41]. Moreover, age is definitely related to menopausal status and these factors are difficult to dissociate, although they tend to be analysed on an individual basis in clinical studies. This is obviously still a controversial subject and no consensus exists to this day on how age influences the risk of developing BM in BC patients.

#### Menopausal status

Menopausal status might also be related to the development of BM in BC patients since oestrogens are essential regulators of bone remodelling, potentially contributing to a fertile microenvironment that might promote BM [42]. Actually in one retrospective British study with 367 patients, older postmenopausal patients were more likely to have bone-only metastasis than bone and visceral metastases (63% versus 43%, p = 0.0002, univariate analysis) [3]. Regarding pre-menopausal patients, there was a significant increase in bone and visceral metastases versus bone-only disease (37% versus 24%, p = 0.009, univariate analysis). Another study, however, failed to show an association between these factors (n = 336; χ2 = 3.162, p = 0.075). Despite the trend, this was a univariate analysis which did not account for confounding factors such as oestrogen receptor (ER) and progesterone receptor (PR) expression [41].

Of note, there is an irrefutable association between menopausal status and age that has to be taken into account when interpreting these studies.

If, in fact, menopausal status proves to be a risk factor for BM, this might be part of the explanation why adjuvant bisphosphonate treatment in early BC seems to have an impact only in postmenopausal women [43].

#### Body Mass Index (BMI)

There seems to be a clear impact of BMI in BC recurrence-free survival and OS with obese patients showing a worse prognosis [44, 45]. The mechanisms explaining this association are not completely clear but seem to relate with higher levels of oestradiol (given the aromatisation of androgens in adipose tissue in postmenopausal women), higher levels of insulin or even other non-biologic mechanisms such as chemotherapy under-dosing or obesity-related complications [46]. Nevertheless, BMI does not seem to relate to the pattern of metastases found in early BC patients who relapsed [45].

#### Histological type

Different invasive BC histological types, namely ductal, lobular, medullar, tubular, and mucinous BC are widely known to show different metastatic patterns [47]. There is however some controversy on whether histological type might relate to the risk of BM, with some studies showing no relationship at all [48] and others showing an association as detailed ahead.

The latest studies suggest a higher likelihood of lobular BC to metastasise to the bone [39, 40, 49]. Nevertheless, while highly significant in univariate analysis, histological type does not significantly correlate with the risk of developing BM in a multivariate model [39]. It has been argued that the association between histological type and metastatic pattern might be related to the fact that most lobular invasive carcinomas are of luminal A and luminal B subtypes, whilst this is less frequent for patients with other histologies [39]. When taking into account other factors known to be implicated in the risk of BM, such as the intrinsic subtype, histological type does not seem to relate with the risk of having BM and lobular histology (at least for the classic variant) might in fact be a surrogate marker for luminal A and luminal B subtypes instead [39].

#### Grade

Several studies relate grade to the risk of developing BC metastasis, regardless of the site. When analysing which risk factors are associated with BM, those that indicate a less aggressive disease are generally referred [41, 48].

As such, BM associate more often with lower-grade primary BC than with tumours showing a lesser degree of differentiation [39, 48]. Of note, this feature can imperfectly select for luminal A tumours.

#### Tumour size

Several authors have reported an effect of tumour size in the risk of developing BM, with bigger tumours showing a higher risk [50, 51]. Wei et al reported that tumours with bone-only metastasis (21 cases) had a mean size of 2.8 cm when compared to tumours without BM which had a mean size of 1.8 cm (p-value not reported). Yamashiro *et al* reported that patients with T2 tumours had a HR of 2.02 (95% CI: 1.385–2.958) for being BM free when compared to patients with T3 (HR = 4.14, 95% CI: 2.506–6.836), or T4 tumours (HR = 6.40; 95% CI: 3.951–10.374). Nevertheless, when considered in a multifactorial analysis, tumour size does not consistently increase the risk of BM in a statistically significant way and rather tumour stage seems to stand out [50]. Also, a more recent study with 9652 female Japanese BC patients failed to show a relationship between tumour size and BM development, even in the univariate analysis [39]. As such, tumour size does not seem to be a major factor for BM development.

#### Lymph node involvement

Lymph node (LN) involvement is a known risk factor for metastasis in BC patients [52]

Similarly to tumour stage, LN involvement was also considered an independent risk for BM in several studies [4, 50, 51]. Nevertheless, other studies do not show a significant relationship between LN metastasis and the risk of BM in BC patients [39]. Although it might be a factor contributing to BM risk, LN metastasis do not seem to play a dominant role when compared to others such as intrinsic subtype [39].

### Genetic factors

Recent access to large datasets of genetic information linked to clinicopathological and outcomes data, as well as the thriving study of cancer biomarkers, enabled the study of genetic associations with the pattern of metastases. Some of these factors are detailed in Table 2.

#### Genetic signatures

a. Intrinsic subtype

It has long been known that BC is a heterogeneous disease which is susceptible to multiple classifications. The classification based on immunohistochemistry, and in particular in the presence of ER and PR, appears to strongly correlate with BM [41, 53].

Another classification system, using prognostic multigene classifiers, classifies BC in five intrinsic subtypes (luminal A, luminal B, HER-2-enriched, basal-like, and normal-like), associated with distinct morphologies and clinical implications [54, 55]. Basal-like tumours have a higher rate of distant nodal, lung, and brain metastases and a lower propensity to generate both bone and liver metastases. Bone is, actually, the least common metastatic site for basal-like tumours [1, 56].

Luminal A subtype is definitely a risk factor for relapse in the bone [56]. Indeed bone seems to be the predominant site of metastasis for luminal subtypes (80.5% of the tumours), while basal-type and HER-2-like tumours showed BM in 41.7 and 55.6% respectively (p = 0.001) [57]. In fact luminal B subtype is more likely to have bone as a first recurrence site when compared to other subtypes (p = 0.005) [58].

Even in studies using HER-2 overexpression, ER and PR status, and Ki67 as surrogate markers for intrinsic subtypes, this results hold true, with bone being the predominant site of metastasis in 66.6% of luminal A-like tumours, 71.4% of the luminal B-like tumours, and 65% of the luminal/HER2-like groups [1, 59]. The St Gallen International Expert Consensus on the Primary Therapy of Early Breast Cancer 2015 endorsed a surrogate classification system that proved to identify BC subtypes as one of the most important risk factors in determining the site of relapse [60, 61].

b. Bone-specific metastasis-related genes

In 2003, Kang *et al* published a study in which BC cell populations with high affinity to bone in mouse models showed a distinct transcriptional signature. In this study, most of the genes that were highly overexpressed in BM (up-regulated more than four-fold) encoded cell membrane or secretory products that might favour metastasis, such as chemokine receptor CXCR4, fibroblast growth factor-5 (FGF-5), connective tissue derived growth factor (CTGF), interleukin-11 (IL-11), follistatin and matrix metalloproteinase, and collagenase MMP1 [62]. On the other hand, a heterogeneous group of genes were downregulated, including extracellular matrix components and receptors (such as laminin β1, fibronectin, collagen type V, integrin β4), cytoskeletal components (tubulin α1, keratin 7, periplakin), proteinases (serpin A1, cathepsin B), class II major histocompatibility complex components (HLA-DPA1, -DPB1, -DPB3), and putative tumour suppressors (N33, DLC1) [62]. Therefore, it was suggested that primary tumour cells need to acquire certain characteristics in order to successfully originate BM. This BM-specific genetic profile would be superimposed to a poor prognosis gene signature [63]. Kang’s study was, however, based in mouse model systems. Other papers confirmed the overexpression of the upregulated genes in human BM with different types of solid tumours [64]. Nevertheless, a subsequent study refuted Kang’s BM signature's ability to discriminate tumours prone to develop BM [65]. However, it allowed distinguishing between primary BC that preferentially metastasised to bone from BC that preferentially metastasised elsewhere. This suggested that the enrichment of the gene signature could allow the prediction of BM in primary BC [65, 66].

A different study using 157 primary breast tumours of patients with known metastatic disease identified 15 genes differentially expressed in tumours with and without BM. This 15-gene BM signature included the upregulation of three genes in the primary tumour–*NAT1*, *BBS1*, and *PH-4* associated with metabolic, protein transport, and oxidation-reduction processes respectively. The other 11 genes were downregulated [57]. These genes mostly encode molecules with protein binding function, many of them being membrane-bound. This signature was afterwards validated using a 376 BC published dataset in which 81.2% of the positive tested tumours had also clinically evident BM. The 15-gene expression signature remained associated with the likelihood of BM development in ER-positive and ER-negative tumour groups [57].

Although some of these studies seem to determine gene signatures that relate with BM, there is a striking lack of overlap amongst them. To this day, no genomic predictor of bone-specific metastasis was clinically validated [66].

### Molecular changes

#### MAF protein overexpression

A recent study described v-maf avian musculoaponeurotic fibrosarcoma oncogene homolog (MAF) as a mediator of BC BM [67]. MAF is a transcription factor that controls the expression of several genes involved in BM such as PTHrP. Using human luminal BC cell lines prone to BM (MCF7, ZR-75 and T47D) and 4T1 murine BC cells, the authors determined copy number aberrations (CNA) frequently present in BC BM. Afterwards, these CNA were examined in independent BC datasets and tested in clinical samples. Results showed that 16q23 gain CNA (which encodes the transcription factor MAF) is associated with BM (HR = 14.5; 95% CI 6.4–32.9; p<0.001); the same association was found between MAF overexpression and BM (HR = 2.5; 95% CI 1.7–3.8; p<0.001). Nevertheless, neither 16q23 gain nor MAF overexpression related to visceral metastases [67].

The relationship between MAF and the risk of BM is thought to result from the control that this protein might exert on PTHrP [67]. Nonetheless, neither of these tests has yet been incorporated in clinical practice as a method of allowing an early detection of BC patients at risk for developing BM.

#### Prolactin receptor

Other hormones besides oestrogen and progesterone seem to relate with BC risk. Prolactin, for example, has been reported to be important in some subpopulations. Indeed, higher serum prolactin levels seem to be related to higher BC risk in postmenopausal women under hormone replacement therapy (HRT), not showing an effect in non-HRT users and even showing an inverse non-significant association in pre-menopausal women [68]. When looking at *in situ* BC, there is a positive association between serum prolactin levels and the risk of disease, regardless of menopausal status or hormone therapy use [69].

Other studies have also shown a possible association between a rise in serum prolactin levels after BC surgery and poorer outcomes in postmenopausal women [70]. When analysing the expression of prolactin receptor (PRLR), higher levels in the primary tumour are seen associated with a shorter time to BM [71]. Also, PRLR is present in the microenvironment of BC BM where it has the potential to further induce osteoclast formation [71].

#### Increased expression of parathyroid hormone-related protein (PTHrP)

PTHrP is a cytokine with high molecular similarity to PTH [72] that shares its receptor [73] and biological activity [74, 75]. About 60% of BC are found to produce PTHrP, along with other types of cancer frequently associated with BM such as prostate and lung cancer [76, 77]. Several studies have associated PTHrP expression to the development of BM in BC patients.

Indeed PTHrP is expressed in 73–100% BC BM [78, 79], a clearly higher frequency to that found in visceral metastases and primary tumours [78].

When considering primary tumours, the expression of PTHrP seems to correlate with the development of BM [80, 81] though the directionality of the association, much like other risk factors, is still controversial.

In one study that included 367 patients with invasive primary BC and after a median follow-up of 67 months (range 3–120 months), PTHrP was detected by immunohistochemistry in 72% of primary tumours [82]. In this study, when analysed in a multivariate model, the absence of expression of PTHrP in the primary tumour was an independent predictor of BM (p = 0.002), together with the number of positive axillary LN (p<0.001), lymphatic/vascular invasion (p = 0.002), and the absence of PR staining (p = 0.04). In fact, the HR for the development of BM in women with PTHrP expressing tumours was 39% of that in BC patients with a PTHrP-negative tumour (95% CI 21–71%, p = 0.002) [82]. Additional analysis of this cohort including 526 patients, with a median follow-up time of ten years, detected PTHrP staining in 79% of primary tumours. After stratification by stage, the HR for BM between BC patients with PTHrP positive and negative tumours was 0.63, favouring a risk reduction of BM in the group of PTHrP positive tumours (95% CI, 0.41–0.98; p = 0.04) [83].

Still, other studies have refuted this view [77, 84]. One of these studies analysed 125 primary tumours and demonstrated PTHrP staining in 63.2%. With median follow-up of 97 months (range 5–243), PTHrP staining in primary tumour was found to correlate with the development of BM with a HR of 7.104 (95% CI, 1.782–48.110; p = 0.0037), along with having a T4 tumour, in a multivariate logistic regression analysis [77].

Several factors might contribute to these contradictory results: the methods used for detecting PTHrP expression as well as the criteria for PTHrP scoring were different between studies with Henderson *et al* considering positive staining if one or more cells were positive. Since there were different recruitment periods (1989–2000 for Henderson *et al*, with more than two thirds of patients recruited before 1996 [83] and 1996–1999 for Takagaki *et al* [77]) along with other potential differences in risk factors for BM, the course of the disease may have been influenced accordingly. Therefore, PTHrP staining in primary tumour is not yet an established risk factor for the development of BM in BC patients, although it might seem pathophysiologically reasonable that it would relate to an increased risk of BM.

#### Increased expression of parathyroid hormone receptor (PTHR)

As previously mentioned, PTHrP shares its receptor with PTH. Following the same rationale that suggests that PTHrP expression in primary tumours might relate to increased BM risk, there are several studies trying to understand the association between PTHR expression in primary tumours and the risk of developing BM.

One of these studies measured the expression of PTHR in primary tumours (n = 67) and BM (n = 13), using reverse transcriptase polymerase chain reaction [85]. Similarly to PTHrP expression, its receptors are also more frequently found in BM when compared to primary tumours, although this was not a statistically significant difference (85% versus 58%, p = 0.053). Moreover, the median receptor expression level was also higher in BM (median: 2818; interquartile range (IQR): 1189–7483) than in primary tumours (median: 299; IQR 10–3000), p<0.05. This may mean that PTHR expression confers a selective advantage for tumour cells in BM.

A more recent study aimed to relate the immunohistochemical expression of specific proteins in primary tumours with the development of BM [86]. For this purpose a total of 184 patients were analysed (n = 113 with BM and n = 71 with extraskeletal metastases) for ER, PR, HER2, Ki67, and six exploratory proteins: COX2, CK5/6, CXCR4, PTHR1, OPN, and CaSR. PTHR1 overexpression was found in 34% of patients with BM compared to 16% without BM (p = 0.007). While there seemed to be a positive relationship between PTHR1 overexpression and BM in the univariate analysis, this did not hold true in the multivariate model where only ER and absence of cytoplasmic OPN were independent risk factors for BM (p = 0.002 and p = 0.018) [86].

As such, although PTHR expression might be a selective advantage for tumour cells to grow or survive in bone, it is not yet clear if the expression of this receptor in primary tumour has any effect in the development of BM.

#### Bone sialoprotein

Bone sialoprotein (BSP) is an important component of the bone extracellular matrix, along with other proteins such as osteocalcin and osteopontin [87, 88].

There are several studies that establish a relationship between BSP expression (determined by immunohistochemistry) in the primary tumour and the risk of developing BM. This was first described in a cohort of 39 BC patients, 22 of whom developed BM during a follow-up time of at least three years, showing that high expression of BSP was associated with the development of BM (p = 0.008, Mann Whitney test) [89]. This is, however, a small study in a very specific population with a particularly high risk of recurrence (22 out of 39 patients had a recurrence in a three-year period). In fact, it lacks a multivariate analysis to exclude potential confounders and, therefore, may not be applicable to all BC patients.

A prospective study with 388 patients with median follow-up time of 20 months used a different technique for BSP detection, employing serum samples in which the protein was detected by a radioimmunoassay [88]. Women with bone-only metastasis showed a higher preoperative median serum BSP level (48.3 ng/mL, range 9.53–152.9) when compared with those with both osseous and visceral metastases (30.6 ng/mL, range 25.5–59.5) and with patients with visceral metastases only (12.3 ng/mL, range 5.5–22.7). BSP value greater than or equal to 24 ng/mL was a significant prognostic factor for the development of BM both in the univariate analysis (RR of 94.10, p<0.001) and in the multivariate analysis (RR 93.96, P<0.001). This association was not present when the analysis was repeated for patients with visceral metastases only (p = 0.38) [88].

There seems to be a correlation between serum BSP levels and the risk of BM in BC patients. The underlying mechanism that explains this remains unclear.

#### Bone morphogenetic protein 7 (BMP7)

Bone morphogenetic proteins are cytokines which owe their name to the ability to participate in the formation of bone. Despite that, they have a multiplicity of effects that range from controlling important steps of embryonic development to regulating growth, differentiation and apoptosis of different cell types [90]. They also seem to have a role in the development of cancer which is still not perfectly understood [91].

Even so, BMP7 expression appears to correlate with BM in BC patients. By analysing primary tumour samples from 483 women with BC (only in 409 protein expression was retrieved), it became clear that tumours expressing BMP7 were more prone to develop BM (20% versus 14%, median follow-up time of 15 years), although this was not a statistically significant difference [91]. More importantly, BMP7 expressing tumours showed an accelerated BM formation (p = 0.04), which held true for invasive ductal carcinomas (p = 0.033) but not for invasive lobular carcinomas (p = 0.29). In the multivariate analysis, BMP7expression was an independent risk factor for accelerated BM formation (HR = 2.14, 95% CI 1.07–4.28; p = 0.032) [91].

BMP7 expression in primary tumour seems to correlate with the risk for accelerated BM formation in BC patients. It is still not currently validated to stratify BM risk.

#### Macrophage-capping protein (CAPG) and PDZ domain-containing protein GIPC1 (GIPC1)

Recently a new composite biomarker for BM in BC has been described. Westbrook *et al* identified potential biomarkers for BM in variants of human BC cells and performed clinical validation in the large cohort of patients of the AZURE study [92]. In this study, authors identified proteins which expression was increased more than two-fold in BC BM cell lines when compared to lung metastatic BC cell lines or non-metastatic BC cell lines. Given that CAPG and GIPC1 showed a higher expression in BM cell lysates, they were selected for clinical validation. This was done in a training set of 427 randomly assigned patients from the AZURE trial (211 control, 216 zoledronate) and in a second independent validation set of 297 randomly assigned patients, also from the AZURE trial (147 control, 150 zoledronate). Analysis of the control arm showed an increased risk for skeletal related events (SRE) in patients with high CAPG and GIPC1 scores. CAPG and GIPC1 bivariate score has been identified and validated as a prognostic biomarker for BM when considering SRE alone (p = 0.011) and SRE and other events (p = 0.037).

Worth noticing that a high bivariate score did not correlate significantly with SRE in patients who received zoledronate [92].

This study concluded that a biomarker including CAPG and GIPC1 in primary BC tissue is associated with BM development. It also correlates with worse OS (five year OS of 76.2% CI 64.4–90.3 when compared to patients with lower bivariate score, five year OS of 85.9%, 95% CI 81.7–90.4; HR 1.81, 95% CI 1.01–3.24, p = 0.045) and predicts adjuvant zoledronate benefit; in the zoledronate arm five year OS was 82.3% (95% CI = 77.6–87.3) for patients with high bivariate score and 88.2% (95% CI = 78.0–99.8) in patients with low bivariate score; (HR = 0.71, 95% CI = 0.32– 1.55, P = 0.385). This suggests a treatment benefit of approximately 2.5-fold [92].

### Markers of bone turnover

#### Bone crosslinked C telopeptide (B-CTx)

The bone has a dynamic metabolism that results from the equilibrium between the activity of osteoblasts and osteoclasts [93]. Bone resorption is therefore a continuous process which leads to the release of certain markers into the circulation. B-CTx is one of these markers and its pretreatment serum concentration has been referred as a possible predictor for BM [93].

In a prospective study of 621 BC patients, 123 had a recurrence. Higher pre-treatment B-CTx was associated with lower bone-only relapse free survival (HR of 3.43; 95% CI 1.20–9.77; p = 0.02) even when analysed with other factors such as tumour size, nodal status, and C-peptide [93]. There was no significant association between tumour size and B-CTx.

### Effect of treatment

There is a profound effect of adjuvant therapy in the pattern of metastasis.

For instance, tamoxifen exposure is known to reduce the risk of BM in ER positive BC patients, and this effect persists after treatment discontinuation [4]. However, most of these studies are retrospective and the heterogeneity found amongst them might be a result of the diversity of the adjuvant regimens used.

For adjuvant chemotherapy, the results are not as clear. When considering the International Breast Cancer Study Group (IBCSG) randomised clinical trials, which included 2018 patients between 1978 and 1985, comparison between patients with node positive disease who received more effective treatments (six or more cycles of cyclophosphamide, methotrexate, fluorouracil and prednisone, with or without tamoxifen or tamoxifen and prednisone alone) with patients who received less effective treatments (no treatment or a single cycle of chemotherapy) denotes a reduction in the risk of loco-regional or distant soft tissue first relapse (from 36% to 18% at ten years, p = 0.0001) in favour of the more effective treatments. Nevertheless, in this study, the risk of bone relapse did not change when more effective treatments were used [94]. This is, however, a study using old chemotherapy regimens and was not analysed taking into account other factors such as tumour intrinsic subtype. In fact, ER status was only known in 52% of the patients [94]. Therefore, this might not be applicable to all tumour subtypes or to the chemotherapy currently used.

The use of anti-HER-2 therapy (with trastuzumab, pertuzumab, lapatinib, or T-DM1) also has an important impact in the natural history of HER-2 positive BC. Taking this into account, there is a general belief that this sort of therapy might change the pattern of metastatic spread [95–97]. Several studies seem to refute this claim. Serpico *et al* suggested that anti-HER-2 treatment of metastatic HER-2 positive patients does not affect the progression of metastatic sites. In this study the risk of progression in each site is particularly high if that organ was already affected by disease [28]. In fact, when analysing the proportion of patients who progressed in each site (visceral, soft tissue, bone, and CNS), heterogeneity along treatment lines was mostly explained by random error (range *I*^2^ index: 0.0–20.3%) [28]. Another retrospective cohort study corroborates this finding, as the first-site of distant relapse did not significantly differ between HER-2 positive patients (n = 303) who had or had not received previous trastuzumab in neoadjuvant and/or adjuvant setting (p = 0.144). In this study, patients without previous trastuzumab had BM on first relapse in 22.3% of the cases when compared to 25.7% of patients who had been previously treated with trastuzumab [98]. When evaluating if adjuvant trastuzumab decreases the risk of a recurrence in a specific site, anti-HER-2 therapy seems to relate with a decrease in the risk of liver recurrence versus other sites (OR = 0.63, 95% CI: 0.41–0.96, p = 0.033) but no other differences in recurrence sites were reported [99].

Other type of therapy which might influence the pattern of metastases is the use of bone modifying agents. Adjuvant bisphosphonate use reduced the risk BM as shown in a meta-analysis of 26 different trials with a total of 18.766 women with early BC enrolled: there was a reduction of risk of BM, risk of fracture, and even a lower BC mortality, although definite benefit was only seen in postmenopausal women [43]. In fact, in the AZURE phase III trial, adjuvant zoledronic acid was associated with a reduction in BM as first or subsequent site of recurrence [100].

Likewise, denosumab addition to adjuvant aromatase inhibitor in postmenopausal patients with early stage hormone receptor positive BC (n = 3425) also improved disease-free survival (DFS). In the ABCSG-18 phase III study, adding denosumab (60mg, every 6 months, subcutaneously) reduced recurrence rate by 18% [101].

## Conclusion

There are several known factors which increase the risk for BC dissemination. Regarding bone-only metastasis several risk factors have been evaluated with conflicting results amongst different studies that in part derived from highly heterogeneous study populations, cancer treatment administered, and study methodology. Nevertheless, intrinsic subtype is widely accepted as a major risk factor for BM development.

There has been some effort in trying to establish other gene-signatures that might correlate with BM development and at least two sets of signatures seem to correlate with increased risk. Other factors such as BMP7 expression, higher level of bone sialoprotein or elevation of B-CTx prior to treatment may also indicate a higher risk for BM.

Progress with cell lines, animal models, and clinical studies will allow a better understanding of the complex invasion-metastasis cascade. Desirably, a more profound knowledge of risk factors for BM should lead to the development of algorithms to determine if each patient has an increased risk for bone disease. This should in turn trigger the use of specific therapies in order to increase bone DFS. Further research on this topic is, therefore, mandatory.

## Conflict of interest

There is no conflict of interest to declare.

## List of abbreviations

BC: breast cancer

BM: bone metastases

BMI: body mass index

BMPs: bone morphogenetic proteins

BMP7: bone morphogenetic protein 7

CAN: copy number aberrations

CAPG: Macrophage-capping protein

CTC: circulating tumour cells

DFS: disease free survival

EMT: epithelial-to-mesenchymal transition

ER: oestrogen receptor

ET-1: endothelin-1

FGF: fibroblast growth factors

GIPC1: PDZ domain-containing protein GIPC1

HR: hazard ratio

HRT: hormone replacement therapy

IGF-1: insulin-like growth-factor-1

LN: lymph node

MAF: v-maf avian musculoaponeurotic fibrosarcoma oncogene homolog

MET: mesenchymal-to-epithelial transition

OPG: osteoprotegerin

OS: overall survival

PDGF-BB: tumour-derived platelet-derived growth factor-BB

PR: progesterone receptor

PRLR: prolactin receptor

PTHrP: parathyroid hormone-related peptide

RANKL: receptor activator of NF-кB ligand

RR: relative risk

SRE: skeletal related event

TGF- β: transforming growth factor β

## Figures and Tables

**Table 1.** Clinicopathological factors related to the development of bone metastasis (BM) in breast cancer (BC) patients.

Risk Factor	Association	References
Age	Unknown/Conflicting results	[4, 39–41]
Menopausal status	Unknown/Conflicting results	[3, 41]
BMI	No relationship	[45]
Histological type	No relationship (surrogate marker for intrinsic subtype)	[39, 40, 48, 49]
Grade	Inverse/Conflicting results	[41, 48]
Tumour size	No relationship	[39, 50, 51]
Lymph node (LN) involvement	Might contribute, not major	[39]

**Table 2.** Genetic and molecular factors, and markers of bone turnover related to the development of BM in BC patients.

Risk Factor		Association	References
**Genetic Factors**			
Intrinsic Subtype		One of the most important risk factors (Luminal subtype linked with BM)	[1, 56, 57, 60]
Bone specific metastasis related genes	102-gene signature	Association with BM	[62, 64]
	15-gene signature	Association with BM	[57]
**Molecular changes**			
MAF protein		16q23 gain and MAF overexpression associated with BM	[67]
Prolactin receptor		Shorter time to BM	[71]
Increase in PTHrP		Unknown/Conflicting results	[77, 83, 84]
Increase in PTHR		Unknown/Conflicting results	[85, 86]
Bone sialoprotein		BSP> 24ng/mL is a significant prognostic factor for bone-only metastasis	[88, 89]
BMP7		Independent risk factor for accelerated BM formation	[91]
Composite biomarker CAPG and GIPC1		Predicts disease outcomes and benefit from zoledronate	[92]
**Markers of bone turnover**			
B-CTx		Shorter time to bone-only metastasis	[93]

## References

[1] Kennecke H (2010). Metastatic behavior of breast cancer subtypes. J Clin Oncol.

[2] Manders K (2006). Clinical management of women with metastatic breast cancer: a descriptive study according to age group. BMC Cancer.

[3] Coleman RE, Smith P, Rubens RD (1998). Clinical course and prognostic factors following bone recurrence from breast cancer. Br J Cancer.

[4] Liede A (2016). The incidence of bone metastasis after early-stage breast cancer in Canada. Breast Cancer Res Treat.

[5] Lee S J (2011). Implications of bone-only metastases in breast cancer: Favorable preference with excellent outcomes of hormone receptor positive breast cancer. Cancer Res Treat.

[6] Ahn SG (2013). Prognostic factors for patients with bone-only metastasis in breast cancer. Yonsei Med J.

[7] Valastyan S (2012). Tumor metastasis: molecular insights and evolving paradigms. Cell.

[8] Xue C (2003). The gatekeeper effect of epithelial-mesenchymal transition regulates the frequency of breast cancer metastasis. Cancer Res.

[9] Wang Y, Zhou B P (2011). Epithelial-mesenchymal transition in breast cancer progression and metastasis. Chin J Cancer.

[10] Han W (2016). Oriented collagen fibers direct tumor cell intravasation. Proc Natl Acad Sci USA.

[11] Streuli CH, Gilmore AP (1999). Adhesion-mediated signaling in the regulation of mammary epithelial cell survival. J Mammary Gland Biol Neoplasia.

[12] Martin SS, Leder P (2001). Human MCF10A mammary epithelial cells undergo apoptosis following actin depolymerization that is independent of attachment and rescued by Bcl-2. Mol Cell Bio.

[13] Mohme M, Riethdorf S, Pantel K (2016). Circulating and disseminated tumour cells — mechanisms of immune surveillance and escape. Nat Rev Clin Oncol.

[14] Nash GF (2002). Platelets and cancer. Lancet Oncol.

[15] Gupta GP, Massagué J (2004). Platelets and metastasis revisited: a novel fatty link. J Clin Invest.

[16] Witz IP (2008). The selectin-selectin ligand axis in tumor progression. Cancer Metastasis Rev.

[17] Fidler IJ (1970). Metastasis: quantitative analysis of distribution and fate of tumour emboli labeled with 125I-5-iodo-2 [prime]-deoxyuridine. J Natl Cancer Inst.

[18] Luzzi KJ (1998). Multistep nature of metastatic inefficiency: dormancy of solitary cells after successful extravasation and limited survival of early micrometastases. Am J Pathol.

[19] Wong CW (2001). Apoptosis: an early event in metastatic inefficiency. Cancer Res.

[20] Chen J (2016). Efficient extravasation of tumor-repopulating cells depends on cell deformability. Sci Rep.

[21] Ito S (2001). Real-time observation of micrometastasis formation in the living mouse liver using a green fluorescent protein gene-tagged rat tongue carcinoma cell line. Int J Cancer.

[22] Broggini T (2016). Passive entrapment of tumor cells determines metastatic dissemination to spinal bone and other osseous tissues. PLoS One.

[23] Azevedo AS (2015). Metastasis of circulating tumor cells: favorable soil or suitable biomechanics, or both?. Cell Adhes Migr.

[24] Paget S (1989). Distribution of secondary growths in cancer of the breast,1889. Cancer Metastasis Rev.

[25] Weilbaecher KN, Guise TA, McCauley LK (2011). Cancer to bone: a fatal attraction. Nat Rev Cancer.

[26] Zhou W (2014). Cancer-Secreted miR-105 destroys vascular endothelial barriers to promote metastasis. Cancer Cell.

[27] Lu X, Kang Y (2017). Organotropism of breast cancer metastasis. J Mammary Gland Bio Neoplasia.

[28] Serpico D (2015). Disease progression pattern in metastatic breast cancer patients treated with anti-HER2 therapies. Clin Transl Oncol.

[29] Hoshino A (2015). Tumour exosome integrins determine organotropic metastasis. Nature.

[30] Ursini-Siegel J, Siegel PM (2016). The influence of the pre-metastatic niche on breast cancer metastasis. Cancer Lett.

[31] Brockton N T (2015). The Breast Cancer to Bone (B2B) Metastases Research Program: a multi-disciplinary investigation of bone metastases from breast cancer. BMC Cancer.

[32] Gunasinghe NP (2012). Mesenchymal-epithelial transition (MET) as a mechanism for metastatic colonisation in breast cancer. Cancer Metastasis Rev.

[33] Thomas R J (1999). Breast cancer cells interact with osteoblasts to support osteoclast formation. Endocrinology.

[34] Mundy GR (2002). Metastasis to bone: causes, consequences and therapeutic opportunities. Nat Rev Cancer.

[35] Du Y (2007). Fusion of metabolic function and morphology: sequential [18F] fluorodeoxyglucose positron-emission tomography/computed tomography studies yield new insights into the natural history of bone metastases in breast cancer. J Clin Oncol.

[36] Yi B (2002). Tumor-derived platelet-derived growth factor-BB plays a critical role in osteosclerotic bone metastasis in an animal model of human breast cancer. Cancer Res.

[37] Yin J J (2003). A causal role for endothelin-1 in the pathogenesis of osteoblastic bone metastases. Proc Natl Acad Sci USA.

[38] Brama M (2007). Osteoblast-conditioned medium promotes proliferation and sensitizes breast cancer cells to imatinib treatment. Endocr Relat Cancer.

[39] Diessner J (2016). Evaluation of clinical parameters influencing the development of bone metastasis in breast cancer. BMC Cancer.

[40] Purushotham A (2014). Age at diagnosis and distant metastasis in breast cancer - A surprising inverse relationship. Eur J Cancer.

[41] Chen J (2013). Analysis of clinicopathological factors associated with bone metastasis in breast cancer. J Huazhong Univ Sci Technolog Med Sci.

[42] Hofbauer LC (2014). Endocrine aspects of bone metastases. Lancet Diabetes Endocrinol.

[43] Breast Cancer Trialists E, Group C (2015). Articles adjuvant bisphosphonate treatment in early breast cancer: meta-analyses of individual patient data from randomised trials. Lancet.

[44] Imkampe AK, Bates T (2010). Impact of a raised body mass index on breast cancer survival in relation to age and disease extent at diagnosis. Breast J.

[45] Yazici O (2015). The effect of obesity on recurrence pattern in early breast cancer patients. J Buon.

[46] Chan DSM (2014). Body mass index and survival in women with breast cancer-systematic literature review and meta-analysis of 82 follow-up studies. Ann Oncol.

[47] Dixon AR (1991). A comparison of the clinical metastatic patterns of invasive lobular and ductal carcinomas of the breast. Br J Cancer.

[48] James JJ (2003). Bone metastases from breast carcinoma: histopathological - radiological correlations and prognostic features. Br J Cancer.

[49] Sastre-Garau X (1996). Infiltrating lobular carcinoma of the breast: Clinicopathologic analysis of 975 cases with reference to data on conservative therapy and metastatic patterns. Cancer.

[50] Yamashiro H (2014). Prevalence and risk factors of bone metastasis and skeletal related events in patients with primary breast cancer in Japan. Int J Clin Oncol.

[51] Wei B (2008). Bone metastasis is strongly associated with estrogen receptor-positive/progesterone receptor-negative breast carcinomas. Hum Pathol.

[52] Colzani E (2014). Time-dependent risk of developing distant metastasis in breast cancer patients according to treatment, age and tumour characteristics. Br J Cancer.

[53] Smid M (2008). Subtypes of breast cancer show preferential site of relapse. Cancer Res.

[54] Sorlie T (2001). Gene expression patterns of breast carcinomas distinguish tumor subclasses with clinical implications. Proc Natl Acad Sci USA.

[55] Perou C M (2000). Molecular portraits of human breast tumours. Nature.

[56] Huber KE, Carey LA, Wazer DE (2009). Breast cancer molecular subtypes in patients with locally advanced disease: impact on prognosis, patterns of recurrence, and response to therapy. Semin Radiat Oncol.

[57] Savci-Heijink CD (2016). A novel gene expression signature for bone metastasis in breast carcinomas. Breast Cancer Res Treat.

[58] Metzger-Filho O (2013). Patterns of recurrence and outcome according to breast cancer subtypes in lymph node-negative disease: Results from international breast cancer study group trials VIII and IX. J Clin Oncol.

[59] Vaz-Luis I, Winer E P, Lin N U (2013). Human epidermal growth factor receptor-2-positive breast cancer: Does estrogen receptor status define two distinct subtypes?. Ann Oncol.

[60] Soni A (2015). Breast cancer subtypes predispose the site of distant metastases. Am J Clin Pathol.

[61] Coates A S (2015). Tailoring therapies-improving the management of early breast cancer: St Gallen International Expert Consensus on the Primary Therapy of Early Breast Cancer 2015. Ann Oncol.

[62] Kang Y (2003). A multigenic program mediating breast cancer metastasis to bone. Cancer Cell.

[63] van ’t Veer LJ (2002). Gene expression profiling predicts clinical outcome of breast cancer. Nature.

[64] Casimiro S (2012). Analysis of a bone metastasis gene expression signature in patients with bone metastasis from solid tumors. Clin Exp Metastasis.

[65] Minn AJ (2005). Distinct organ-specific metastatic potential of individual breast cancer cells and primary tumors. J Clin Invest.

[66] Casimiro S (2016). Molecular mechanisms of bone metastasis: which targets came from the bench to the bedside?. Int J Mol Sci.

[67] Pavlovic M (2015). Enhanced MAF oncogene expression and breast cancer bone metastasis. J Natl Cancer Inst.

[68] Tikk K (2014). Circulating prolactin and breast cancer risk among pre- and postmenopausal women in the EPIC cohort. Ann Oncol.

[69] Tikk K (2015). Circulating prolactin and in situ breast cancer risk in the European EPIC cohort: a case-control study. Breast Cancer Res.

[70] Wang D (1986). Serum prolactin levels in women with breast cancer and their relatopnship to survival. Eur J Clin Oncol.

[71] Sutherland A (2016). The role of prolactin in bone metastasis and breast cancer cell – mediated osteoclast differentiation. J Natl Cancer Inst.

[72] Suva LJ (1987). A parathyroid hormone-related protein implicated in malignant hypercalcemia: cloning and expression. Science.

[73] Abou-Samra AB (1992). Expression cloning of a common receptor for parathyroid hormone and parathyroid hormone-related peptide from rat osteoblast-like cells: a single receptor stimulates intracellular accumulation of both cAMP and inositol trisphosphates and increases intracel. Proc Natl Acad Sci.

[74] Diel IJ (2001). Prognostic factors for skeletal relapse in breast cancer. Cancer Treat Rev.

[75] Horiuchi N (1987). Similarity of synthetic peptide from human tumor to parathyroid hormone in vivo and in vitro. Science.

[76] Southby J (1990). Immunohistochemical localization of parathyroid hormone-related protein in human breast cancer. Cancer Res.

[77] Takagaki K (2012). Parathyroid hormone-related protein expression, in combination with nodal status, predicts bone metastasis and prognosis of breast cancer patients. Exp Ther Med.

[78] Vargas SJ (1992). Localization of parathyroid hormone-related protein mRNA expression in breast cancer and metastatic lesions by in situ hybridization. J Bone Miner Res.

[79] Linforth R (2012). Coexpression of parathyroid hormone related protein and its receptor in early breast cancer predicts poor patient survival. Clin Cancer Res.

[80] Bundred NJ (1992). Parathyroid hormone related protein and skeletal morbidity in breast cancer. Eur J Cancer.

[81] Guise TA (1997). Parathyroid hormone-related protein and bone metastases. Cancer.

[82] Henderson MA (2001). Parathyroid hormone related protein production by breast cancers, improved survival, and reduced bone metastases. J Natl Cancer Inst.

[83] Henderson MA (2006). Parathyroid hormone-related protein localization in breast cancers predict improved prognosis. Cancer Res.

[84] Kohno N (1994). The expression of parathyroid hormone-related protein in human breast cancer with skeletal metastases. Surg Today.

[85] Hoey RP (2003). The parathyroid hormone-related protein receptor is expressed in breast cancer bone metastases and promotes autocrine proliferation in breast carcinoma cells. Br J Cancer.

[86] Winczura P (2015). Immunohistochemical predictors of bone metastases in breast cancer patients. Pathol Oncol Res.

[87] Zhang JH (2004). Bone sialoprotein promotes bone metastasis of a non-bone-seeking clone of human breast cancer cells. Anticancer Res.

[88] Diel IJ (1999). Serum bone sialoprotein in patients with primary breast cancer is a prognostic marker for subsequent bone metastasis. Clin Cancer Res.

[89] Bellahcene A (1996). Bone sialoprotein expression in primary human breast cancer is associated with bone metastases development. J Bone Min Res.

[90] Boon MR (2011). Bone morphogenetic protein 7: A broad-spectrum growth factor with multiple target therapeutic potency. Cytokine Growth Factor Rev.

[91] Alarmo EL (2008). Bone morphogenetic protein 7 expression associates with bone metastasis in breast carcinomas. Ann Oncol.

[92] Westbrook JA (2016). CAPG and GIPC1: Breast cancer biomarkers for bone metastasis development and treatment. J Natl Cancer Inst.

[93] Lipton A (2011). Elevated bone turnover predicts for bone metastasis in postmenopausal breast cancer: Results of NCIC CTG MA.14. J Clin Oncol.

[94] Goldhirsch A (1994). Effect of systemic adjuvant treatment on first site of breast cancer relapse. Lancet.

[95] Swain SM (2015). Pertuzumab, trastuzumab, and docetaxel in HER2-positive metastatic breast cancer. N Engl J Med.

[96] Krop IE (2014). Trastuzumab emtansine versus treatment of physician’s choice for pretreated HER2-positive advanced breast cancer (TH3RESA): A randomised, open-label, phase 3 trial. Lancet Oncol.

[97] Geyer CE (2006). Lapatinib plus capecitabine for HER2-positive advanced breast cancer. N Engl J Med.

[98] Lambertini M (2015). Patterns of care and clinical outcomes of first-line trastuzumab-based therapy in HER2-positive metastatic breast cancer patients relapsing after (Neo) adjuvant trastuzumab: an Italian multicenter retrospective cohort study. Oncologist.

[99] Vaz-Luis I (2012). Impact of hormone receptor status on patterns of recurrence and clinical outcomes among patients with human epidermal growth factor-2-positive breast cancer in the National Comprehensive Cancer Network: a prospective cohort study. Breast Cancer Res.

[100] Coleman R (2012). Adjuvant zoledronic acid in patients with early breast cancer: Final efficacy analysis of the AZURE (BIG 01/04) randomised open-label phase 3 trial. Lancet Oncol.

[101] Gnant M (2016). Abstract S2-02: The impact of adjuvant denosumab on disease-free survival: Results from 3,425 postmenopausal patients of the ABCSG-18 trial. Cancer Res.

